# The Sensor-Based Physical Analogue Scale as a Novel Approach for Assessing Frequent and Fleeting Events: Proof of Concept

**DOI:** 10.3389/fpsyt.2020.538122

**Published:** 2020-11-26

**Authors:** Stefan Stieger, Irina Schmid, Philip Altenburger, David Lewetz

**Affiliations:** Department of Psychology and Psychodynamics, Karl Landsteiner University of Health Sciences, Krems an der Donau, Austria

**Keywords:** experience sampling method (ESM)/ecological momentary assessment (EMA), wearable devices, cyber ostracism, digital phenotyping, indirect assessment

## Abstract

New technologies (e.g., smartphones) have made it easier to conduct Experience Sampling Method (ESM) studies and thereby collect longitudinal data *in situ*. However, limiting interruption burden (i.e., the strain of being pulled out of everyday life) remains a challenge, especially when assessments are frequent and/or must be made immediately after an event, such as when capturing the severity of clinical symptoms in everyday life. Here, we describe a wrist-worn microcomputer programmed with a Physical Analogue Scale (PAS) as a novel approach to ESM in everyday life. The PAS uses the position of a participant's forearm between flat and fully upright as a response scale like a Visual Analogue Scale (VAS) uses continuous ratings on a horizontal line. We present data from two pilot studies (4-week field study and lab study) and data from a 2-week ESM study on social media ostracism (i.e., when one's social media message is ignored; *N* = 53 participants and 2,272 event- and time-based assessments) to demonstrate the feasibility of this novel approach for event- and time-based assessments, and highlight advantages of our approach. PAS angles were accurate and reliable, and VAS and PAS values were highly correlated. Furthermore, we replicated past research on cyber ostracism, by finding that being ignored resulted in significantly stronger feelings of being offended, which was more pronounced when ignored by a group compared to a single person. Furthermore, participants did not find it overly difficult to complete the assessments using the wearable and the PAS. We suggest that the PAS is a valid measurement procedure in order to assess fleeting and/or frequent micro-situations in everyday life. The source code and administration application are freely available.

## Introduction

The Experience Sampling Method (ESM)—that is, the collection of longitudinal data from participants in their everyday lives—has not only contributed to psychologists' understanding of how people behave in the real world (1), but has also enhanced understanding in other disciplines, such as psychiatry [see special issue (2)] and economics (3). Relative to cross-sectional and laboratory studies, ESM reduces recall bias, provides temporally-dense profiles of each participant, and results may be more externally valid because they capture psychological phenomena in participants' natural environments. Smartphones and wearable microcomputers have recently made it easier to conduct ESM studies (4, 5). However, limiting interruption burden (i.e., the strain of being pulled out of everyday life) remains a challenge, especially when participants must make frequent and/or immediate assessments, such as when capturing the severity of symptoms in everyday life [i.e., (5, 6)]. High interruption burden might result in low compliance, which results in missing data, which in turn may introduce measurement error and selection bias, i.e., data quality decreases and the advantages of ESM studies vanish. Here, we describe how a wearable microcomputer programmed with a Physical Analogue Scale (PAS) can be used to assess fleeting and/or frequent events in everyday life. We utilize data from two pilot studies and an ESM study on social media ostracism to demonstrate the advantages of our approach for reliable and accurate event- and time-based assessments.

### Experience Sampling Method With Smartphones and Wearables

Smartphones are now commonly used in ESM studies (7, 8), with wearable microcomputers (typically worn on the wrist) also increasingly used [e.g., (5, 9, 10); for a review, see (11)]. Although smartphones and wearables have a number of advantages over traditional paper-and-pencil diaries [e.g., the possibility of timestamping data, (12)], limiting interruption burden remains a challenge. Participants are sometimes unwilling to go through the onerous process of removing and unlocking a smartphone, opening the application, carrying out the assessment, closing the application, turning off the smartphone, and replacing it; that is, their commitment may sometimes dramatically decrease when they have to respond very frequently (9). Furthermore, participants may sometimes find it difficult to comply with lengthy procedures (e.g., in job situations; while driving), resulting in delayed assessments.

Relative to smartphones, wearables offer shorter access time (13), are more comfortable (14), and allow researchers to use more reliable tactile vs. auditory signals (10). However, many existing wearables are still rather bulky, expensive, and need to be recharged frequently (15). Some wearables only work in combination with a smartphone (e.g., many commercially-available smartwatches) and use proprietary software. Furthermore, their small displays make it difficult to use text-based instructions or response scales.

### A Novel Approach: The Sensor-Based Physical Analogue Scale

**Figure 1:**
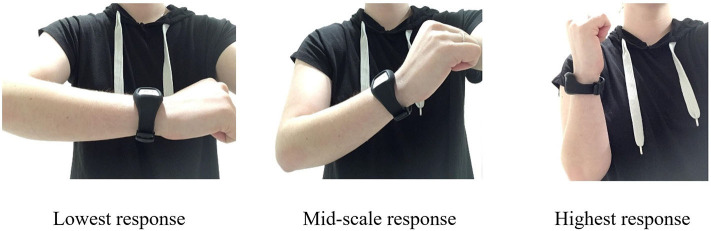
The physical analogue scale.

To address the disadvantages of existing approaches and take advantage of sensor-based data (16), we programmed a wrist-worn wearable with a PAS. The PAS uses the position of a participant's forearm as a continuous response scale. Specifically, participants indicate a response by positioning their forearm flat (0° = lowest scale value), in a fully upright position (90° = highest scale value), or somewhere in-between (see Figure 1). Participants press the wearable's button to record their response, at which point the built-in accelerator sensor determines, timestamps, and locally stores the angle. The PAS thus makes it possible to quickly and intuitively conduct assessments without questionnaires or visual response scales. Because interruption burden is low, the PAS also makes it possible to assess even very fleeting phenomena and/or conduct very frequent assessments, which may be especially useful for clinical psychologists and psychiatrists, for whom symptoms could be assessed longitudinally rather than requiring memory of symptomatology in anamnesis (6). Because the PAS also allows for continuous measurement, it can be compared to Visual Analogue Scales (VAS), which are psychometric response scales where participants indicate a position on a graphically presented continuous line between two end-points.

### Validating the Physical Analogue Scale: First Pilot Studies

In the first pilot study, we were interested in how well people are able to estimate a certain angle. With VAS, people can usually easily estimate, say, the middle of the scale, e.g., the middle of a graphically presented line. But how good are people when instructed to estimate the middle of a 90° angle? In an ESM field study (4 weeks duration), we instructed participants to estimate 45° when prompted by the wearable using a time-based sampling procedure with a haptic prompt.

In the second pilot study, we were interested in whether a PAS measurement comes to the same result as a VAS measurement. In a laboratory study, we asked participants to judge their extraversion using eight items, first in an online questionnaire using a VAS and later doing the same assessment using the wearable and the PAS.

### Using the Physical Analogue Scale to Assess Fleeting and Frequently-Occurring Phenomena: The Example of Social Media Ostracism

To demonstrate both the feasibility of our approach for reliable and accurate event- and time-based assessments and the advantages of the wearable/PAS approach, we conducted an ESM study on the effects of social media ostracism (17). Many social media platforms now integrate a so-called “seen-function” for outgoing messages in their software, which signals to users when a recipient has seen their message (e.g., WhatsApp uses a gray tick-mark to indicate when a message has left the sender's device, two gray tick-marks to indicate when the message has been delivered, and two blue tick-marks once the recipient has seen the message). Knowing that someone has seen but not responded to a message (i.e., social media ostracism) may cause the sender to experience negative emotions, such as feeling ostracized and/or offended (18). Given the current permanently-online, permanently-connected *zeitgeist* (19), social media ostracism represents a frequently-occurring daily life event with immediate effects. Although social media ostracism occurs in people's everyday lives (18, 20, 21), thus far almost all studies on its effects have either been conducted in the laboratory or are cross-sectional [for exceptions, see (22, 23)]. Social media ostracism is thus an ideal micro-situation for showcasing the advantages of the wearable/PAS method.

Our main aim was to demonstrate the feasibility of our approach for reliable and accurate event- and time-based assessments and the advantages of the wearable/PAS method: if the method is valid, we should be able to replicate the results of previous research. Based on existing research (20, 22, 24), we had two hypotheses:

**Hypothesis 1: People feel offended after experiencing social media ostracism (compared to their own personal baseline)**.

**Hypothesis 2: People feel more offended when a group vs. a single recipient ignores a message**.

We also analyzed the extent to which feeling offended generally (i.e., participants' personal baselines) was associated with several personal characteristics. We expected that feeling offended would be positively correlated with Neuroticism, narcissism, and perceived text message dependency, and negatively correlated with self-esteem (22) and collective self-esteem related to online groups (CSE-OG). We had no specific hypotheses about the relationships between feeling offended and the personality traits of Agreeableness, Conscientiousness, Openness to Experience, and Extraversion; their inclusion was purely exploratory (for study preregistration, see https://osf.io/7j3e9/).

## Methods—First Pilot Study

### Participants and Procedure

Eight subjects (75% female; *M*_age_ = 33.3, *SD*_age_ = 9.59, range = 21–49 years) participated in this study. Six subjects used the wearable on their left arm and the other two on the right one (i.e., mostly the non-dominant hand). Data collection started on the same day for each participant and ended after 4 weeks. Participants were instructed to hold the forearm at an angle of 45° and press the button once whenever they were signaled by a haptic stimulus elicited by the wearable itself (time-based sampling procedure). During the data collection phase, participants were in diverse field settings, ranging from the office to leisure activities, such as 2-week hiking trips and journeys abroad.

### Wearable

We developed software for a commercially available, openly programmable wearable from mbientlab (MMR+ wristband kit including microcomputer, eight MB memory, re-chargeable battery, case, elastic band, and coin vibrator motor: ~100$; https://mbientlab.com/metamotionr/). Participant data were stored on the wearable and uploaded at the end of the study onto the researchers' smartphone or tablet via a Bluetooth connection (without Internet). Although an integrated infrastructure with servers, databases, and administration interfaces would allow data to be processed in near real-time (25), we elected to use a different approach that does not require additional data security measures (e.g., firewalls, encryption). The wearable had one button and several built-in sensors (e.g., light intensity, acceleration, air pressure, gyroscope). For this study, only the button and the acceleration sensor were enabled and used. The source code and administration application (Android) is freely available (see Open Practices Section). We used the following configuration, which represents a time-based ESM study with three time-points per day. Random signal time points within the following time frames: 8 a.m. to 11 a.m., 11 a.m. to 2 p.m., 2 p.m. to 5 p.m.

### Wearable: Estimate 45° (Dependent Measure)

When participants pressed the wearable's button, the built-in accelerator sensor determined, timestamped, and stored its position in 3-dimensional space, and also saved the number of button presses^1^. The values for x-, y-, and z- were then transformed into an angle between 0° and 90° using the following formula:^2^

$$\text{Angle }\left[\text{degree}\right]=\arctan\;\left(\frac{\left|y\right|}{\sqrt{\left(x^{2}+z^{2}\right)}}\right)*\frac{180}{\pi }$$

## Results—First Pilot Study

**Figure 2:**
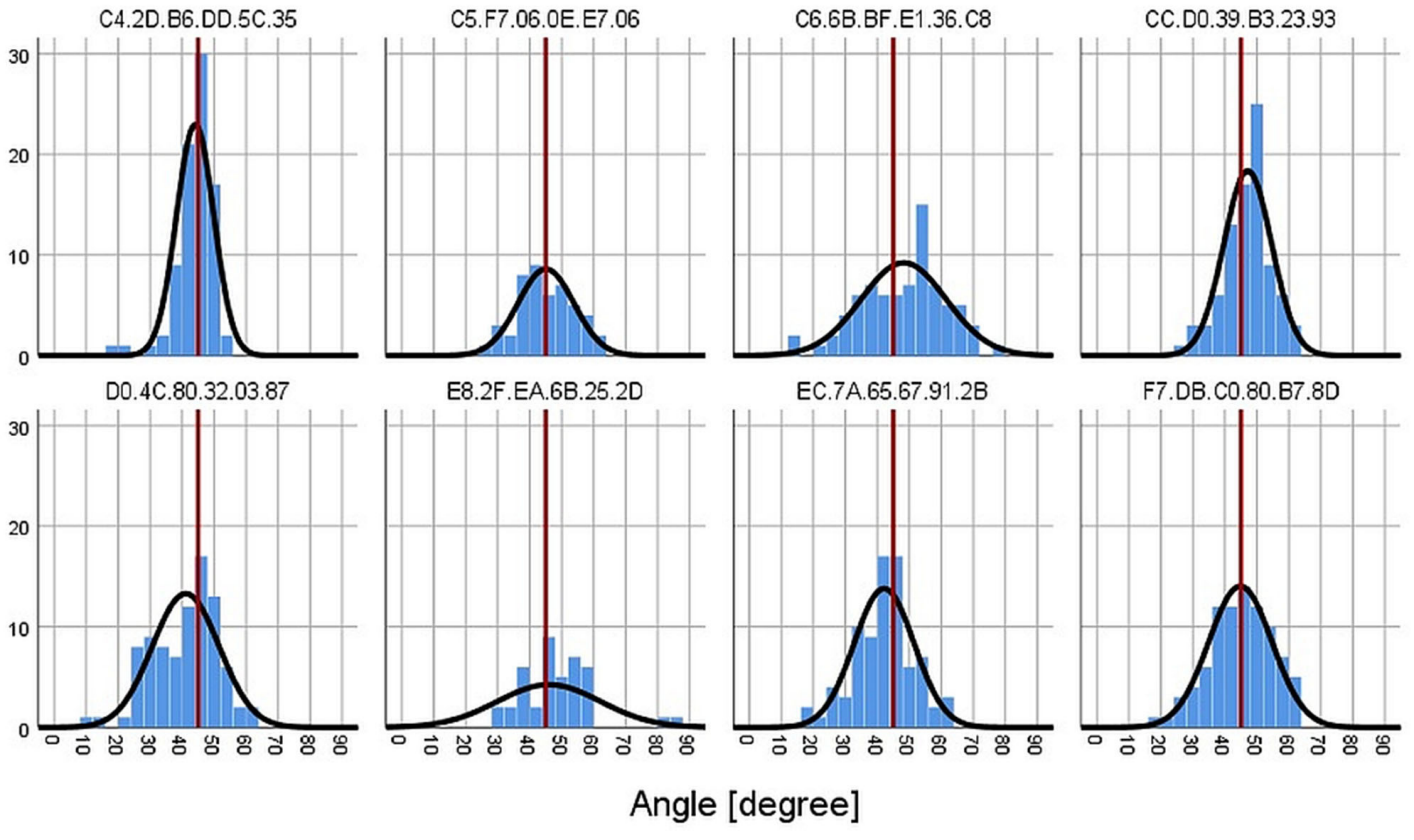
Frequency histogram of all estimated 45° angles for each participant (represented by the wearable's MAC address). The red line marks the 45° from the instruction.

The mean angle over all measurements (*n* = 592) was 44.7° on average (*SD* = 10.38, Median = 45.3; one-sample *t*-test: *t* = −0.75, *p* = 0.454, reference value = 45). The mean angle across participants ranged from 41.1° to 48.2° (range of *SD* = 5.85–16.18). In general, participants were quite accurate in estimating the angle of 45° during the field phase of 4 weeks (see Figure 2), bearing in mind that the field settings were very diverse (from office settings to leisure activities like hiking).

## Methods—Second Pilot Study

### Participants and Procedure

Sixteen subjects (87.5% female; *M*_age_ = 22.7, *SD*_age_ = 2.6, range 20–28 years) participated in this study. Fourteen subjects used the wearable on their left arm and the other two on the right one (i.e., mostly the non-dominant hand). Data collection was realized as a group administration in a classroom. In addition to some test measurements that are not part of this study, participants completed the Extraversion subscale of the BFI (26) on a smartphone using a VAS and in parallel using the PAS on the wearable. For the PAS, participants were instructed to hold the forearm in the desired angle and press the button once. A short haptic feedback was elicited by the wearable when the angle was successfully saved.

### Material–Big Five Inventory [BFI: German Version (26)]

To keep the validation study short, we only assessed the Extraversion subscale (8 Items) of the BFI. For the smartphone administration, we used a VAS (0: *does not apply at all*, 100: *applies very well*) and for the wearable administration the PAS (0°: *does not apply at all*, 90°: *applies very well*).

### Statistical Analyses

We used SPSS (v. 26) to conduct all statistical analyses. We calculated Cronbach α, Pearson correlations, and curve estimation regression analyses to check for linearity between VAS and PAS.

## Results—Second Pilot Study

**Figure 3:**
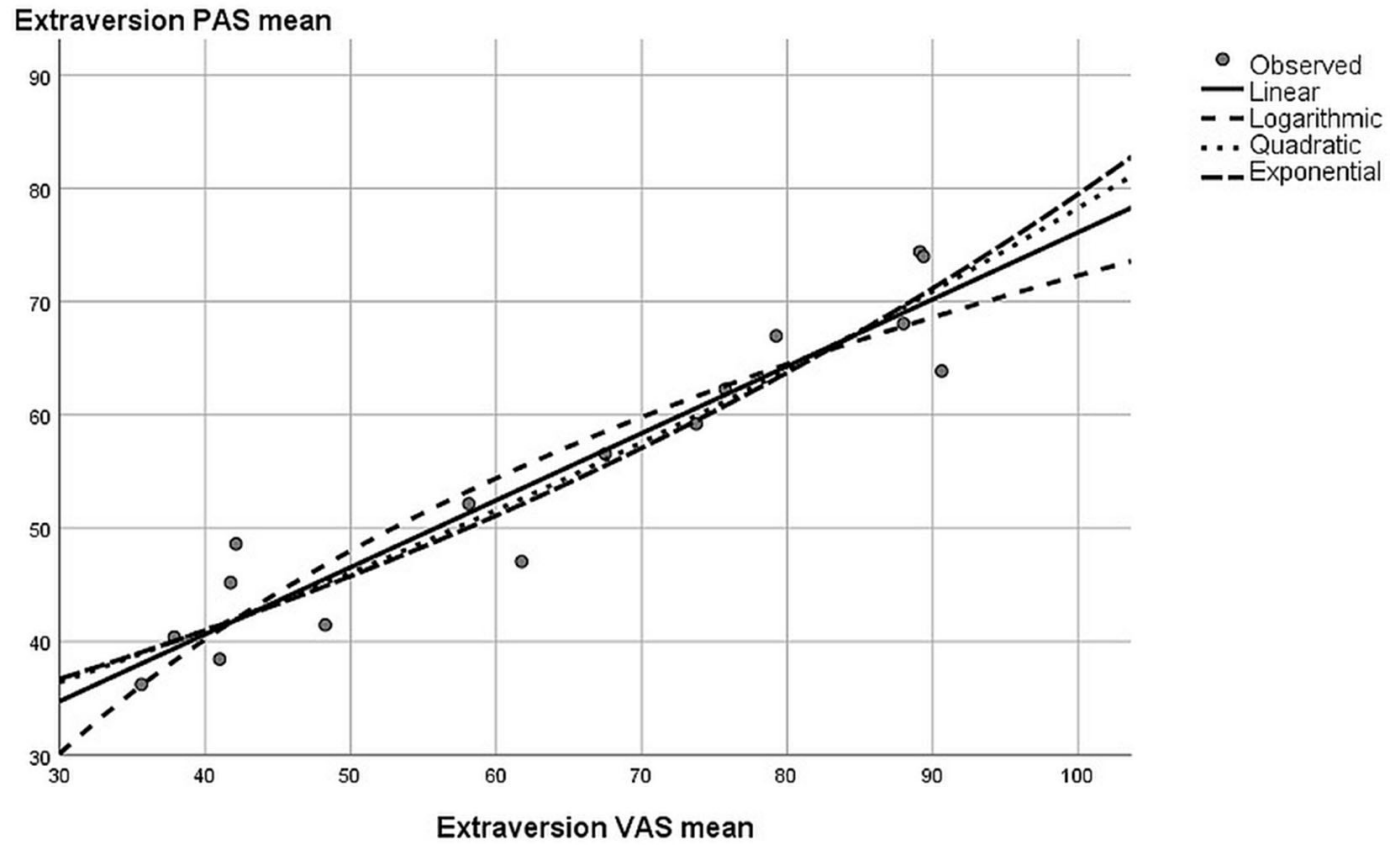
Graphical results from the curve estimation regression—second pilot study.

All extraversion items were highly intercorrelated between the PAS and VAS (*r*s between 0.63 and 0.94, for more details and descriptives, see Supplementary Table 1). Furthermore, reliabilities were all very good (Cronbach α: α_VAS_ = 0.93; α_PAS_ = 0.83) but descriptively slightly lower for the PAS. Extraversion mean scores of the VAS and PAS were highly correlated (*r* = 0.95, *p* < 0.001). To analyze if the relationship between PAS and VAS scores was linear, we calculated a curve estimation regression analysis. It could be that participants when using the PAS were better at differentiating at lower PAS angles but coarser at higher angles (lower angles might be easier to establish than higher angles were the forearm is in an almost upright position). If this was the case, the relationship between PAS and VAS should not be linear. A curve estimation regression did not find substantial differences between linear (*R*^2^ = 0.907), logarithmic (*R*^2^ = 0.886), quadratic (*R*^2^ = 0.910), and exponential (*R*^2^ = 0.905) curve estimations regarding their explained variance levels (see also Figure 3).

## Methods—Ostracism Study

### Participants and Procedure

The research project was conducted in February through April 2019 and participants were recruited on a rolling basis. Based on a power analysis for multi-level designs [(27), p. 123ff], we determined that a minimum sample size of 23 participants with 14 observations each would be sufficient for revealing medium-sized effects of social media ostracism on offendedness [based on (24), we conservatively assumed an effect size of 0.3; α = 5%]. To account for drop-out, technical problems, and so forth, we aimed to recruit 60 participants.

We used a project homepage and a detailed information sheet to provide interested individuals with information about the study objectives and design. Participants were met face-to-face in order to provide them with a fuller description of the assessment procedures. After providing consent, research assistants handed out a wearable that could be easily worn on the wrist. Participants were asked to familiarize themselves with the wearable by clicking once and then waiting for the haptic confirmation (i.e., vibration), then twice, and finally three times. The principal investigators directly answered any questions. Participants also received a credit card-sized laminated information sheet with visual instructions for the PAS, a definition of feeling offended as the variable to be assessed (“Feeling offended: the experience that one's honor, values, and/or feelings having been disregarded or violated, and especially the feeling that one has been insulted”; definition of *Kränkung* from the German-language Wikipedia), and the researchers' contact information. Although the wearable's battery lasts up to 4 weeks, we instructed participants to charge the battery weekly with a USB charger.

Participants then wore the wearable in their everyday lives for 14 days. We collected both event-sampling and time-based sampling data. First, participants were instructed to press the button whenever a single recipient or a group had seen but not responded to their social media message, provided that the participant expected a response. Participants then used the PAS to indicate how offended they felt (event-based sampling). In addition, in order to assess how offended participants generally felt, participants used the PAS to indicate how offended they felt twice every day (time-based sampling). Specifically, the wearable was programmed to issue a vibration signal once between 10 a.m. and 4 p.m. and again sometime between 4 p.m. and 10 p.m., at which point participants used the PAS to indicate how offended they felt at that time. These assessments were used as a baseline measure (i.e., how offended they normally felt during the day). To submit a response, participants positioned their forearm to the appropriate position (from 0° = *not at all offended* to 90° = *extremely offended*) and then pressed the button: once when their message had been ignored by a single recipient, twice when their message had been ignored by a group, and three times for the time-based baseline assessments.

After the field phase, participants returned to the lab and completed an online questionnaire on a laptop computer. Participants entered the first four digits of the wearable's unique media access control address^3^ (printed on the case) so that questionnaire data could be matched with the wearable data while preserving anonymity. After completing the questionnaire, participants deposited the wearable into a box. Participants were thanked and debriefed, and those who wanted to be included in the raffle were asked to provide an email address.

We recruited a total of 59 participants from our social networks. To be eligible for participation, participants had to: (1) own a smartphone, (2) use social media (e.g., WhatsApp, Facebook, Twitter, Snapchat, etc.), and; (3) already use or be willing to activate the “seen function” for outgoing messages for the duration of the study. Six participants (10.2%) dropped out before the end of the study or refused to complete the final questionnaire. Reasons for the drop-out were assessed orally (e.g., unable to use wearables at work, infrequent use of social media, and lost wearables). The final sample size thus consisted of *N* = 53 participants.

Participants in the final sample were *M* = 26.5 years old (*SD* = 9.56, range = 18–57), predominantly women (81%), and from Austria. Most had completed secondary school (63.5%) or had a tertiary degree (26.3%) as their highest level of completed education. Participants were treated in accordance with the World Medical Association Declaration of Helsinki and with local ethical guidelines. They gave informed consent prior to participating. As an incentive, participants could voluntarily enter a raffle with the chance of winning two prizes worth 100 Euro each.

#### Wearable: Feeling Offended (Dependent Variable)

When participants pressed the wearable's button, the built-in accelerator sensor determined, timestamped, and stored its position in 3-dimensional space, and also saved the number of button presses. The values for x-, y-, and z- were then transformed into an angle between 0° (*not at all offended*) and 90° (*extremely offended*).

#### Online Questionnaire Measures

Measures are described in the order of their presentation in the questionnaire.

##### Demographics

Participants reported their age, sex (female/male/other), and highest level of completed education (categories).

##### Social Media Use

Participants responded to four items about their social media use during the field phase: “How many messages have you read in the last 14 days per day on average?,” “How many messages have you sent in the last 14 days per day on average?,” “For how many minutes per day on average have you passively used social media (e.g., reading tweets and Facebook posts, looking at Snapchat pictures, watching YouTube videos)?” and “For how many minutes per day on average have you actively used social media (e.g., writing posts)?” Underlining was used to stress the difference in item wording.

##### Interruption Burden

Participants indicated how difficult it was to conduct the assessments (1: *not at all difficult*, 9: *very difficult*) and how often they forgot to submit a rating over the 2-week period.

##### Self-Esteem

We used the German-version (28) of the Rosenberg Self Esteem Scale [RSES; (29)] to assess participants' global self-esteem. Participants used a 4-point scale (1: *totally disagree*, 4: *totally agree*) to respond to 10 items. Answers to the 10 items were averaged (α = 0.85).

##### Big Five Personality Traits

We used the German-version (26) of the BFI as in the second pilot study (30) to assess participants' personality traits. Participants used a 7-point scale (1: *totally disagree*; 7: *totally agree*) to respond to 44 items. Responses to items related to each trait were averaged (Neuroticism: 8 items, α = 0.63; Extraversion: 8 items, α = 0.80; Conscientiousness: 9 items, α = 0.69; Agreeableness: 9 items, α = 0.69; Openness: 10 items, α = 0.77).

##### Perceived Text Message Dependency

We used the Self-perception of Text-message Dependency Scale (31) to assess the extent to which participants perceived themselves as being psychologically dependent on receiving text messages. Scores on the original scale have adequate psychometric properties (32, 33). We translated the scale into German using the parallel blind technique (34). Participants used a 7-point scale (1: *totally disagree*; 7: *totally agree*) to respond to 15 items about three dimensions of text message dependency: Emotional Reaction (5 items, α = 0.83, e.g., “I feel disappointed if I don't get a reply to my message immediately”), Excessive Use (5 items, α = 0.85, e.g., “I consider myself a quick typist on mobile phones”), and Relationship Maintenance (5 items, α = 0.81, e.g., “I feel disappointed if I don't receive any text messages”). In the current study, we analyzed only overall scores (α = 0.68) to reduce the number of predictors included in the model (for intercorrelations between the subscales, see Supplementary Table 2).

##### Narcissism

We used a short version of the Narcissistic Admiration and Rivalry Questionnaire (35) to assess narcissism. Scores on the scale are reliable and valid (36). Participants used a 6-point scale (1: *not agree at all*, 6: *agree completely*) to indicate their agreement with six items. Scores on the scale can be computed to reflect overall narcissism score or two subdimensions of narcissism (35). Because the reliability for scores on one of the subdimensions was low (Admiration: α = 0.62; Rivalry dimension: α = 0.41), we analyzed only overall scores (α = 0.68).

##### Collective Self-Esteem Related to Online Groups

We used a translated and modified version of the Collective Self-Esteem scale [CSE; (37)] to assess participants' CSE-OG. We first translated the CSE into German using the parallel blind technique (34). We then modified the scale so that all items referred specifically to “online social groups” as opposed to “social groups” in general (i.e., CSE-OG). Participants used a 7-point scale (1: *strongly disagree*; 7: *strongly agree*) to respond to 16 items (e.g., “I am a worthy member of the online social groups I belong to”). Scores on the CSE can be calculated in terms of an overall or in terms of four subdimensions of collective self-esteem. Because the reliability of one subdimension of the adapted scale was low (Importance to Identity: α = 0.45; reliability of all other subdimensions ≥ 0.62), we analyzed only overall scores (α = 0.80).

##### General Comments and Comments About the Wearable

At the end of the online questionnaire, participants had the option of providing open comments (“Do you have any general comments about this wearable study?;” “Do you have comments about the wearable itself, e.g., the signals, which were sent out twice a day using vibration, and so forth?”). For results see, Supplementary Material.

### Statistical Analyses

We used *R* [package lme4 (38), sjstats (39)] to conduct all statistical analyses (40). After a first inspection of the data, we did not exclude any participants even if participation in the longitudinal part stopped before the end of the study. First, we analyzed descriptive statistics (e.g., *M, SD*) and intercorrelations of all study variables. Next, we used a random-intercept, random-slope multi-level regression analysis to analyze the effect of social media ostracism (by either a single-recipient or a group) on how offended participants felt. The multi-level model accounts for the nested design of our study with measurement occasions (level 1) nested within persons (level 2). We created dummy variables for sex (female = 1, male = 2), being ignored by a single recipient (*Single-chat*), and being ignored by a group (*Group-chat*). We ran a baseline model without any predictors to determine the overall intraclass correlation (ICC, i.e., the extent to which how offended participants felt varied between people as opposed to across measurement occasions). We similarly calculated ICCs as indicators of test-retest reliability (i.e., the consistency of responses across measurement occasions; see Supplementary Material).

We then ran a model in which age, sex, self-esteem, Extraversion, Openness, Neuroticism, Agreeableness, Consciousness, text message dependency, narcissism, and CSE-OG were all simultaneously entered on level 2 [all were grand-mean centered except sex; (41)]. The saturated model is displayed below:

Level 1 (within person): Offendedness_ti_ = π_0i_ + π_1i_ Single-chat_ti_ + π_2i_ Group-chat_ti_ +*e*_ti_

Level 2 (between people): π_0i_ = β_00_ + β_01_ Sex_i_ + β_02_ Age_i_ + β_03_ Self-Esteem_i_ + β_04_ Extraversion_i_ + β_05_ Neuroticism_i_ + β_06_ Openness_i_ + β_07_ Agreeableness_i_ + β_08_ Consciousness_i_ + β_09_ Text-message Dependency_i_ + β_010_ Narcissism_i_ + β_011_ CSE-OG_i_ + *r*_0i_

Level 2: π_1i_ = β_10_ + *r*_1i_

Level 2: π_2i_ = β_20_ + *r*_2i_

We used Ω^2^–a generalized *R*^2^ for linear mixed effect models (42)—as a measure of explained variance, with Ω^2^ ≥ 0.01, 0.09, and 0.25, respectively, indicating small, medium, and large shares of explained variance, respectively.

## Results—Ostracism Study

**Figure 4:**
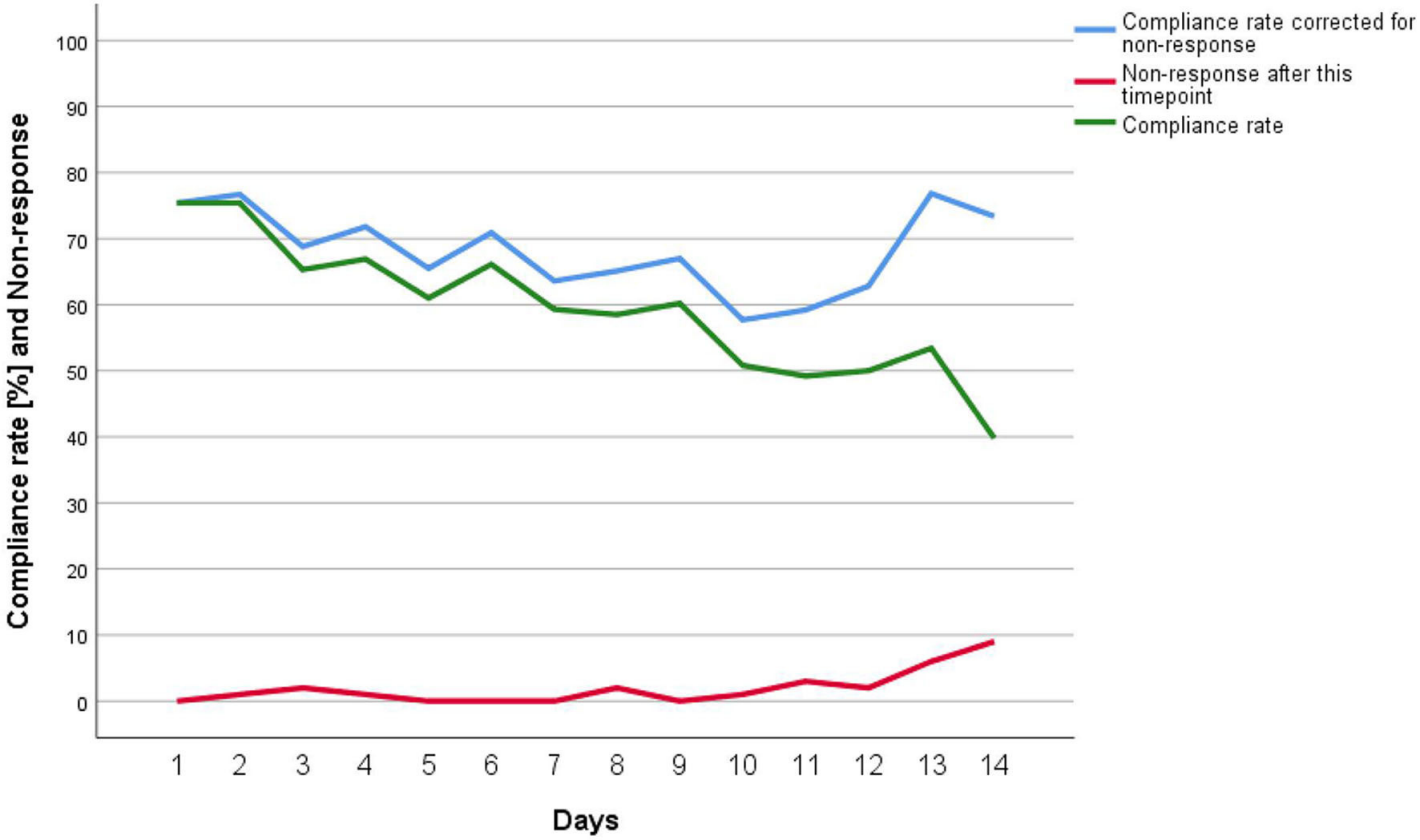
Compliance rate of the time-based assessments and non-response before end of study.

A total of 2,588 responses were recorded (i.e., the angle from the accelerometer in combination with a single-, double-, or triple-button press). We deleted test responses (see Participants and Procedure section) as well as 55 (2.1%) responses that were followed by more than three button presses^4^. This resulted in a final sample of 2,272 assessments, of which 1,031 were time-based (triple-button press) and 1,241 followed an event of social media ostracism (991 times by a single recipient, i.e., single-button press; 250 times by a group, i.e., double-button press). The compliance rate (i.e., whether participants responded to the time-based assessment signals)^5^ dropped slightly over time (*M* = 59.4%; range: 39.8–75.4%; see Figure 4), whereas the final days showed the largest drop, probably due to participants erroneously assuming that the study had ended (i.e., non-response; from 53.4% on day 13 to 39.8% on day 14—the last day of study). If we correct the compliance rate by non-response, the drop in compliance rate is less steep. Drop-out attrition (i.e., leaving the study before the end) was 10% (*n* = 6) and no non-response attrition occurred (i.e., taking part in the study but not pressing the button). Supplementary Table 2 displays the variable intercorrelations and Supplementary Figure 1 displays the distribution of all responses (angles) separated by category (time-based, message ignored by a group, message ignored by a single-recipient). Both the event- and time-based PAS responses were highly consistent across measurement occasions (ICCs > 0.91, see Supplementary Material).

### Interruption Burden

Participants did not find it difficult to conduct the assessments (*M* = 2.4, *SD* = 1.9, Median = 2, range: 1–9; possible scale range: 1–9). Participants estimated that they forgot to submit *M* = 3.8 assessments (*SD* = 3.4; Median = 3; range: 0–15) during the 14-day data collection phase.

### Social Media Use

During the 14 days of data collection, participants reported using social media actively for *M* = 41 minutes (*SD* = 30.8, Median = 30, range: 1–120) and passively for *M* = 80 minutes (*SD* = 65.3, Median = 60, range: 1–240) every day. They read *M* = 62 (*SD* = 137.8, Median = 30, range: 3–1,000) and wrote *M* = 34 (*SD* = 46.5, Median = 20, range: 2–300) social media messages each day. Thus, participants estimated that they wrote a total of 25,144 messages within the 14-day timeframe, implying that ~4.9% or one out of twenty of their messages was ignored.

### People Feel More Offended After Experiencing Social Media Ostracism

The ICC for the null model was 8.9%, indicating that 8.9% of the observed variance in how offended participants felt was associated with differences between people, while 91.1% of the variance was associated with within-person differences across measurement occasions.

Table 1 displays the results of the multi-level analysis. The overall mean level of offendedness (intercept) was 13.5°. Participants felt significantly more offended when a single recipient ignored their message (7.0° more than their own baseline) and even more offended when they were ignored by a group of recipients (11.5° more than their own baseline). The difference between the increase in how offended participants felt after their message was ignored by a group vs. single-recipient was also significant (see Supplementary Table 3)^6^. This supports Hypothesis 1, as well as Hypothesis 2. In sum, the included predictors explained a substantial proportion of variance in how offended participants felt (Ω^2^ = 0.21).

**Table 1:** Results of the multi-level analysis.

**Dependent variable: offendedness predictors**	**Fixed**						**Random**	
	**Coeff**.	**β**	**95% CI**	***B***	***SE***	***t***	**Coeff**.	***SD***
Intercept	β_00_			13.5	0.79	17.01^***^	*r*_0*i*_	3.29
**Within-person**								
Single chat	β_10_	0.20	0.13–0.26	7.0	1.13	6.21^***^	*r*_1*i*_	5.43
Group chat	β_20_	0.21	0.14–0.27	11.5	1.85	6.18^***^	*r*_2*i*_	8.88
**Between-person**								
Sex	β_01_	−0.06	-0.15–0.02	−2.7	1.83	−1.48		
Age	β_02_	0.07	-0.02–0.17	0.1	0.09	1.53		
Self-esteem	β_03_	−0.03	-0.12–0.07	−1.1	1.95	−0.58		
Extraversion	β_04_	−0.01	-0.09–0.09	−0.8	1.07	−0.07		
Neuroticism	β_05_	0.01	-0.10–0.12	0.3	1.37	0.25		
Openness	β_06_	−0.01	-0.09–.07	−0.3	1.13	−0.26		
Agreeableness	β_07_	−0.01	-0.11–0.09	−0.3	1.38	−0.25		
Consciousness	β_08_	> −0.01	-0.11–0.09	−0.2	1.46	−0.14		
Text message dependency	β_09_	0.07	-0.06–0.20	1.1	1.11	1.03		
Narcissism	β_010_	−0.03	-0.15–0.09	−0.7	1.38	−0.51		
CSE-OG	β_012_	−0.02	-0.13–0.08	−0.6	1.29	−0.46		

*All level 2 variables were grand mean centered except for sex. CI, Confidence Interval; CSE-OG, Collective self-esteem related to online groups*.

*** *p < 0.001*.

### Between-Person Differences

None of the level 2 personal characteristics were significantly related to how offended participants generally felt (i.e., across measurement occasions). In follow-up analyses, we also checked whether any of the level 2 variables moderated the increase in how offended participants felt after experiencing social media ostracism (i.e., whether any of the level 2 variables predicted the *slope* of either the level 1 dummy variables “single-chat” or “group-chat”). Again, none of the interactions was significant (for details, see R-code in the online repository).

## General Discussion

Wearables programmed with a PAS have the potential to improve the assessment of frequently occurring and/or fleeting events in participants' everyday lives (5, 9, 10), which may be especially useful in clinical psychology and psychiatric settings where symptoms could be assessed longitudinally (6). In the first pilot study, we showed that participants could accurately estimate an angle of 45° using the PAS in a 4-week field setting. In the second pilot study (lab setting), we confirmed the validity of the PAS by comparing mean extraversion values between the PAS and VAS. Furthermore, the PAS had also comparable reliability to the VAS when assessing extraversion and both formed linear relationships. This suggests that differences in angles are probably equidistant along the measurement scale. Finally, we used data from an ESM study on social media ostracism as an example of a micro-situation that can be difficult to assess in laboratory settings or with traditional cross-sectional questionnaires, but feasible with wearables and the PAS. The wearable/PAS approach worked well. We successfully replicated past research on ostracism (21–23), which found negative effects on emotional states, belongingness (24), and heightened negative affect [for a review, see (43)]. In the present study, we also found negative effects of ostracism, i.e., being ignored online led to feelings of being offended in one-to-one chat situations (Hypothesis 1) and more so when ignored by a group (group chat: Hypothesis 2). Furthermore, our findings of a negative effect of ostracism are in line with other ESM studies on ostracism (22), although still, ESM research is rare (22, 23). Participants also did not find it difficult to complete the assessments and general comments suggested that most participants felt positive or at least neutral about the usage of a wearable (see also the results to the wearable-specific open questions in the Supplementary Material). Nevertheless, compliance rates gradually dropped during the study, with lowest compliance on the last day (see Figure 4). Future research needs to analyze the reasons for this in more detail. In the present study, it may have been an effect of the tactile vibration alarm (e.g., frequency and duration of vibration) or other problems (e.g., time-based signals too early or too late for some participants; see results in Supplementary Material).

Test-retest consistency of subsequent button presses (i.e., two- and three- button presses) was high. Although we did not investigate whether this extrapolates to button-presses with more time in between, this means that the sensors' measurement accuracy was high and, furthermore, that participants did not substantially change the angle of their forearm when pressing the button more than once. Although we investigated validity in rather small sample sizes, the findings suggest that usage of the PAS is feasible, well-accepted by participants, and easy-to-use (5, 9, 10).

Our main focus was on demonstrating the feasibility of our approach for reliable and accurate event- and time-based assessments and advantages of the wearable/PAS approach. Nevertheless, our results also make some important contributions to clinical research on ostracism. By using an ESM design, we were able to assess how often participants experienced social media ostracism in their everyday lives. We found that approximately every 20^th^ message was ignored, causing our participants to feel offended several times a day. Given that people use social media all around the world (~65 billion messages are currently sent each day), the impact of social media ostracism may be a highly relevant experience for people around the globe. It therefore seems worthwhile to further analyze the short- and long-term consequences of social media ostracism.

Interestingly, offendedness differed predominantly within as opposed to between participants, and we found no evidence that personal characteristics (e.g., self-esteem, Big Five traits, text message dependency) explained differences in how offended people generally felt. This does not mean, however, that personal characteristics are completely unrelated to experiences of social media ostracism. Personal characteristics might, for example, matter more in the longer- than in the short-term [e.g., participants with high emotional stability might immediately feel offended by social media ostracism just like their peers, but might return to their baseline level faster; (22)]. Future studies on how the effects of social media ostracism unfold over time would be fruitful [for a similar approach to well-being, see (44)].

### Potentials and Limitations of the Physical Analogue Scale and Other Sensor-Based Data Collection Procedures

With low interruption burden, long battery life, smartphone independence, and relatively low price (~100$), our wearable/PAS approach overcomes several of the challenges associated with previous data collection procedures. Although further validation studies are needed such as the accuracy of the sensors or study compliance in comparison with smartphones, we believe that the wearable/PAS approach offers not only psychologists and psychiatrists but also researchers in other disciplines (e.g., medicine, sociology) a valuable combination for studying micro-events in everyday life (e.g., clinical symptoms). It is another example of how computer science can extend the methods of other sciences, such as psychology (45) or physiology (5). At present, the wearable/PAS can only be used to assess a few items; however, applications could potentially be developed so that additional items could be presented on the touchscreen of existing smartwatches [e.g., using Android Wear; for example, see (5, 10)].

We see large potential for sensor-based scales like the PAS. We think that sensor-based scales are particularly well-suited to capture frequent and/or short-lived phenomena because of the low interruption burden. Furthermore, due to the unobtrusive assessment procedure of the PAS, we think our approach is suitable for the assessment of sensitive topics (e.g., sexuality, racism, suicidal thoughts, self-harming behavior like “cutting”). Aside from that, other sensor-based assessments could be developed in the future, such as using hand tilts as a response scale (46) or the acceleration with which one punches one's own fist into one's open hand as an intuitive measure of aggression. Of course, wearables and sensor-based data do not replace but rather complement more traditional methods. Furthermore, our approach probably will not work for every population and should be thoroughly thought out when planning a study based on the wearable/PAS approach. For example, Vega et al. (4) found, that paper/pencil diaries worked better than several digital measurement procedures in a sample of patients with Parkinson's disease.

### Conclusion

Although further in-depth validation studies are needed, wearables might offer researchers the possibility of delving into participants' everyday lives more deeply than ever before (5, 6, 10) by being unobtrusive and inconspicuous. We have described how an inexpensive wearable programmed with the PAS can be used to assess frequent and/or fleeting events, supplementing past wearable developments. Our validity studies and application of the PAS suggest that the sensor-based PAS is an intuitive, easy-to-use scale for collecting data on how people feel and behave in the real world.

## Data Availability Statement

De-identified data along with the analysis scripts and all materials are posted at https://osf.io/7j3e9/.

## Ethics Statement

Ethical review and approval was not required for the study on human participants in accordance with the local legislation and institutional requirements. The participants provided their written informed consent to participate in this study.

## Author Contributions

SS, IS, and PA developed the ostracism study concept. Testing and data collection were performed by IS and PA for the ostracism study. SS and DL collected the data for the pilot studies and developed the wearable concept. DL programmed the wearable and gave technical support. SS performed the data analysis and interpretation. SS drafted the manuscript and IS, PA, and DL provided critical revisions. All authors contributed to the study design and approved the final version of the manuscript for submission.

## Conflict of Interest

The authors declare that the research was conducted in the absence of any commercial or financial relationships that could be construed as a potential conflict of interest.

## Footnotes

^1^For technical specifications, see https://mbientlab.com/metamotionr/.

^2^We assumed that participants would wear the wearable on the wrist of their non-dominant hand. However, some participants did not or changed the wearable from one hand to another during the data collection phase. We therefore had to use the absolute value of *y*, which effectively mirrors negative angles into positive angles.

^3^A media access control address is a unique identifier assigned to a network interface controller, such as a Bluetooth network device. Because wearables had a Bluetooth connection, each had a unique address.

^4^Some participants indicated difficulty feeling the resistance of the button. To reduce this error, we have adapted the software so that participants receive haptic feedback after every button press.

^5^Compliance rate = (responded to time-based signals / scheduled time-based signals) × 100.

^6^When taking the sign of the angle positive/ negative into account, the mean baseline level of offendedness is lower and the effect sizes of being ignored by a single-recipient and group are higher than reported here.

## Open Practices

The study was preregistered; the preregistration can be assessed at https://osf.io/76kru. De-identified data along with the analysis scripts and all materials are posted at https://osf.io/7j3e9/.

Source code of the administration app can be found on GitHub at https://github.com/KL-Psychological-Methodology/ESM-Board-Admin/.
