# Supplementary material for: The Sensor-Based Physical Analogue Scale as a Novel Approach for Assessing Frequent and Fleeting Events: Proof of Concept
Source: Front Psychiatry. 2020 Nov 26;11:538122. doi: 10.3389/fpsyt.2020.538122 (PMC7732659; doi:10.3389/fpsyt.2020.538122)
Supplement: Supplementary file 1 [file Data_Sheet_1.docx]

Supplementary Materials

# Data Processing

The wearable created two files for each participant. Both files were automatically named based on the wearable’s MAC address. One file included the number of button presses with a timestamp and the second file included the angle of each response, also including a timestamp. Using the MAC address and timestamp as the key variables, the two files were merged and controlled for plausibility.

# Test-Retest Reliability – Ostracism Study

We calculated the mean angles for the subsets of responses followed by two and three button-presses. Responses followed by two button-presses were highly consistent across measurement occasions (Cronbach α = .967; Intraclass correlation coefficient (ICC) [two-way mixed-effects model with absolute-agreement definition] = .936 [.919, .950]) and no mean difference (*F* [1,500] = 0.02, *p* = .884, η_p_^2^ < .001), as were responses followed by three button-presses (*F* [2,3090] = 0.04, *p* = .957, η_p_^2^ < .001; α = .968; ICC = .909 [.900, .918]).

# Wearable-specific Open Questions – Ostracism Study

Nineteen people wrote a general comment and 22 a wearable-specific comment at the end of the online questionnaire. With the general comments, regarding the wearable, three comments were negative (e.g., would prefer smartphone over wearable; uncomfortable to wear and no visual feedback about the battery status; did not feel the vibration alarm), 11 comments were neutral (e.g., general aspects of the study), and five were positive (e.g., wearable worked perfectly; wearable more practical than smartphone; easy handling and always with you; very simple functioning). The wearable-specific comments could be categorized in 4 broad categories: 2 comments about the general design of the study, 17 comments about the vibration alarm, 4 about the handling, and 3 about the button. The vibration alarm was mostly criticized because of the time-frame (e.g., too early in the morning, too late in the evening) but seldom about the haptic feeling (e.g., did not feel the vibration). The handling was questioned because of a missing feedback function about how often the button had been pressed^[[1]](#footnote-1)^, the comfort when wearing it, the look, and the procedure when charging the battery of the wearable (in order to uncover the plug-in, the wearable had to be temporarily removed from its rubber casing). The button itself was only mentioned because of the very flat design and the size.

**Supplementary Tables**

**Table S1**. *Descriptive results of VAS and PAS of second pilot study.*

|  | VAS | PAS | Correlation VAS - PAS |
| --- | --- | --- | --- |
|  | *M* (*SD*) | *M* (*SD*) | *r* |
| Item 1 | 61.4 (24.9) | 53.0 (15.5) | .82*** |
| Item 2 | 72.0 (21.1) | 56.9 (13.8) | .63** |
| Item 3 | 58.8 (19.8) | 48.5 (14.0) | .77*** |
| Item 4 | 72.9 (21.4) | 59.4 (18.5) | .88*** |
| Item 5 | 58.9 (21.3) | 51.4 (16.9) | .82*** |
| Item 6R | 40.4 (33.2) | 35.3 (22.6) | .94*** |
| Item 7R | 53.2 (31.2) | 43.0 (23.5) | .93*** |
| Item 8R | 20.5 (23.7) | 23.6 (22.6) | .90*** |

Note. ** *p* < .01, *** *p* < .001. VAS range 0 to 100, PAS range 0 to 90. VAS = Visual Analogue Scale, PAS = Physical Analogue Scale.

**Table S2**. *Pearson correlations between Level 2 variables of the ostracism study*.

|  | 1. | 2. | 3. | 4. | 5. | 6. | 7. | 8. | 9. | 10. | 11. |
| --- | --- | --- | --- | --- | --- | --- | --- | --- | --- | --- | --- |
| 1. Sex |  |  |  |  |  |  |  |  |  |  |  |
| 2. Age | .05 |  |  |  |  |  |  |  |  |  |  |
| 3. Extraversion | -.10 | -.02 |  |  |  |  |  |  |  |  |  |
| 4. Neuroticism | -.04 | -.18 | -.22 |  |  |  |  |  |  |  |  |
| 5. Openness | .08 | -.04 | .30* | .02 |  |  |  |  |  |  |  |
| 6. Agreeableness | -.14 | .08 | -.04 | -.41** | -.07 |  |  |  |  |  |  |
| 7. Consciousness | -.08 | .30* | .32* | -.09 | .09 | .12 |  |  |  |  |  |
| 8. TMD Emotional Reaction | .20 | -.04 | .03 | .38** | -.12 | -.16 | .15 |  |  |  |  |
| 9. TMD Excessive Use | .07 | -.32* | .14 | .24 | .17 | .04 | .18 | .50*** |  |  |  |
| 10. TMD Relationship Maintenance | .20 | -.37** | -.15 | .20 | -.09 | -.05 | .16 | .42** | .37** |  |  |
| 11. Narcissism | .27† | -.06 | .18 | .36** | .31* | -.50*** | .07 | .47*** | .38** | .33* |  |
| 12. CSE-OG | .12 | .06 | .08 | -.24† | .14 | .13 | .30** | .24† | .28* | .29* | .14 |

*Note*. *N* = 53, Sex: 1 = female, 2 = male, TMD = Text message dependency, CSE-OG = Collective self-esteem related to online groups.

*** *p* < .001, ** *p* < .01, * *p* < .05, † *p* < .10.

**Table S3**. *Results of the multi-level analysis with singe-chat as the reference of the ostracism study.*

| Dependent variable: Offendedness  Predictors | Fixed | | | | | |  | Random | |
| --- | --- | --- | --- | --- | --- | --- | --- | --- | --- |
|  | Coeff. | β | 95% *CI* | *B* | *SE* | *t* |  | Coeff. | *SD* |
| Intercept | β_00_ |  |  | 21.2 | 1.03 | 19.56*** |  | *r*_0_*_i_* | 6.33 |
| Within-person |  |  |  |  |  |  |  |  |  |
| Group chat | β_10_ | .08 | .04 – .13 | 4.4 | 1.43 | 3.07** |  | *r*_1_*_i_* | 4.34 |
| Baseline | β_20_ | -.20 | -.26 – -.14 | -7.1 | 1.05 | -6.78*** |  | *r*_2_*_i_* | 5.09 |

*Note*. CI = Confidence Interval.

** *p* < .01, *** *p* < .001.

**Supplementary Figures**

**Figure S1.** *Histogram of responses to the Physical Analogue Scale separated by category. Bold lines represent the mean angles including a 95% confidence interval of the ostracism study.*

| 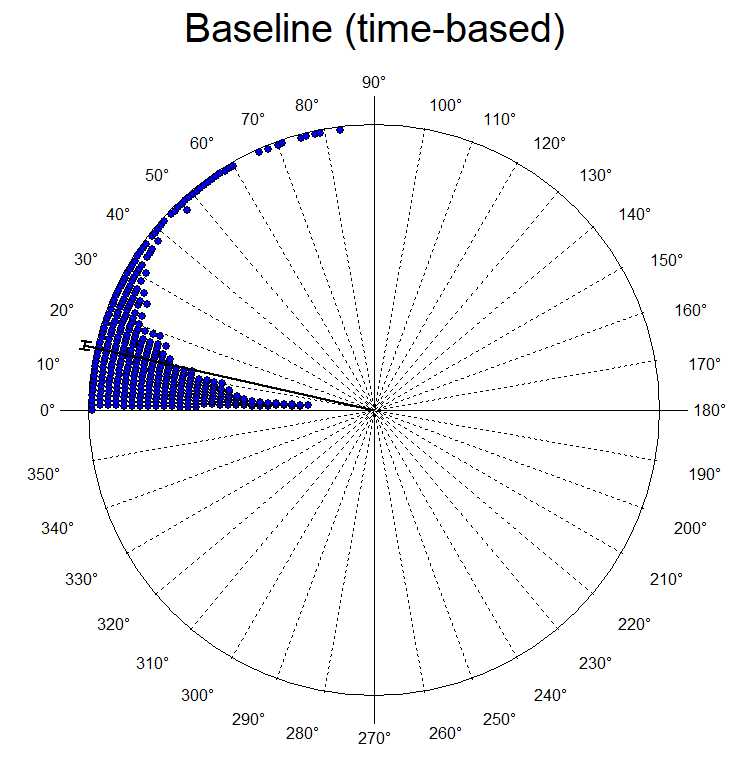 | 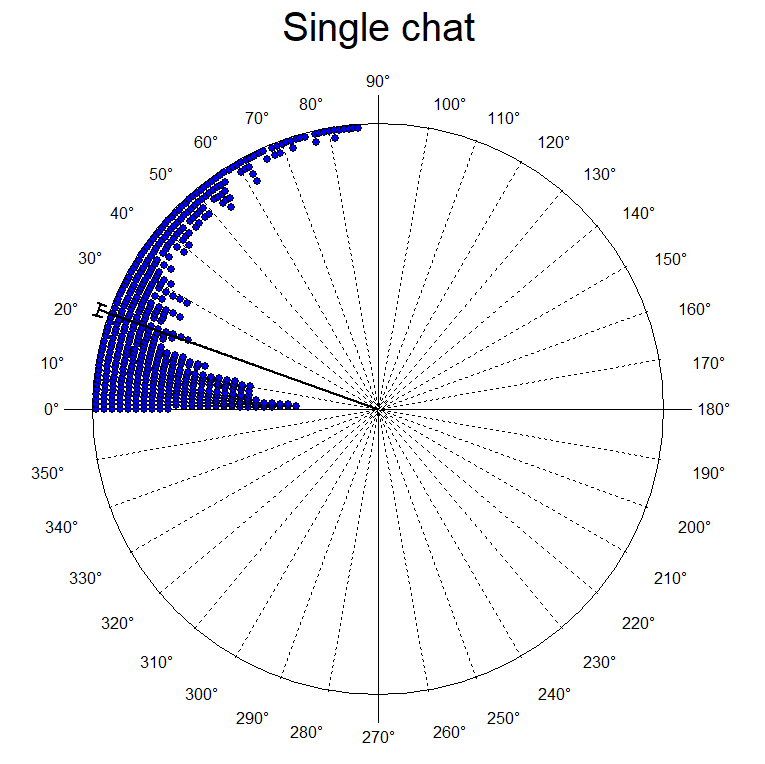 | 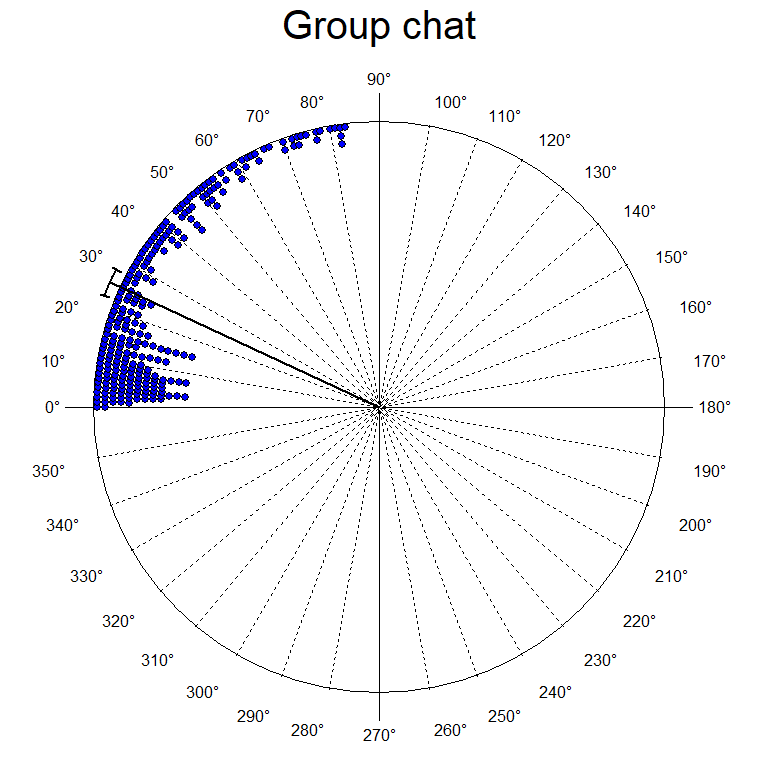 |
| --- | --- | --- |

1. Meanwhile, to reduce this error, we have adapted the software so that participants receive haptic feedback after every button press. [↑](#footnote-ref-1)
